# Accelerated Partial Breast Irradiation: Macrophage Polarisation Shift Classification Identifies High-Risk Tumours in Early Hormone Receptor-Positive Breast Cancer

**DOI:** 10.3390/cancers12020446

**Published:** 2020-02-14

**Authors:** Sören Schnellhardt, Ramona Erber, Maike Büttner-Herold, Marie-Charlotte Rosahl, Oliver J. Ott, Vratislav Strnad, Matthias W. Beckmann, Lillian King, Arndt Hartmann, Rainer Fietkau, Luitpold Distel

**Affiliations:** 1 Department of Radiation Oncology, Universitätsklinikum Erlangen, Friedrich-Alexander-Universität Erlangen-Nürnberg, Universitätsstraße 27, D-91054 Erlangen, Germany; soeren.schnellhardt@fau.de (S.S.); marie.rosahl@gmail.com (M.-C.R.); Oliver.ott@uk-erlangen.de (O.J.O.); vratislav.strnad@uk-erlangen.de (V.S.); rainer.fietkau@uk-erlangen.de (R.F.); 2 Institute of Pathology, Universitätsklinikum Erlangen, Comprehensive Cancer Center Erlangen-EMN, Friedrich-Alexander-Universität Erlangen-Nürnberg, Krankenhausstraße 8-10, D-91054 Erlangen, Germany; Ramona.erber@uk-erlangen.de (R.E.); Arndt.Hartmann@uk-erlangen.de (A.H.); 3 Department of Nephropathology, Institute of Pathology, Universitätsklinikum Erlangen, Friedrich-Alexander-Universität Erlangen-Nürnberg, Krankenhausstraße 8-10, D-91054 Erlangen, Germany; Maike.Buettner-Herold@uk-erlangen.de; 4 Department of Gynecology and Obstetrics, Universitätsklinikum Erlangen, Comprehensive Cancer Center Erlangen-EMN, Friedrich-Alexander-Universität Erlangen-Nürnberg, Universitätsstraße 21, D-91054 Erlangen, Germany; fk-direktion@uk-erlangen.de; 5 Intensive Care Unit, Redcliffe Hospital; University of Queensland, 4072 Brisbane, Queensland, Australia; Lillian.jy.king@gmail.com

**Keywords:** tumour associated macrophages, CD68, CD163, prognosis, early breast cancer, hormone receptor-positive, accelerated partial breast irradiation

## Abstract

Studies have demonstrated correlations between accumulations of tumour-associated macrophages (TAMs), especially of M2-like phenotype, and increased mortality in advanced breast cancer. We investigated the prognostic potential of both main macrophage phenotypes in early hormone receptor-positive (HR+) breast cancer. The studied cohort of 136 patients participated in an institutional APBI phase II trial. Patient selection was characterized by HR+, small tumour size and no metastasis. Tissue microarrays from pre-RT resection samples were double stained for CD68/CD163 using immunohistochemistry. CD68+/CD163− cells were considered M1-like macrophages and CD68+/CD163+ was representative of M2-like macrophages. M1 and M2 macrophage densities were analysed semi-automatically in the stromal and intraepithelial tumour compartment. Low M1 and high M2 densities were strongly associated with decreased disease-free survival (DFS). Combined TAM phenotype densities were studied after defining a macrophage shift classification: M1-shifted (M1 high, M2 low) and non-shifted (M1 low, M2 low; M1 high, M2 high) tumours entailed a favourable outcome. In contrast, M2-shifted (M1 low, M2 high) TAM populations were associated with extremely reduced DFS. Thus, the full predictive potential of TAMs was revealed in a combined analysis of both phenotypes. The M2-shifted subgroup of tumours is classified as high-risk and probably not suitable for partial breast irradiation.

## 1. Introduction

Breast cancer is the most commonly diagnosed malignancy in women [1]. Medical advances and increased public awareness have enabled earlier diagnosis and treatment of breast cancer in developed countries [2,3]. Early breast cancer diagnosis is generally associated with a good prognosis; however, given its high incidence there is still a significant number of relapses [4,5,6]. Thus, the development of new prognostic tools for early cases is equally as important as for those with unfavourable prognosis.

A promising subset of prognostic biomarkers are tumour-associated macrophages (TAMs). According to a commonly utilised model there are two main phenotypes of macrophages, M1 and M2. M1 polarised macrophages primarily engulf and destroy pathogens with reactive oxygen and nitrogen species. In contrast, M2 macrophages remove cell debris and initiate repairs in their surroundings through the release of growth factors like EGF, VEGF and TGF-β. M1 and M2 macrophages cooperate in the control and execution of local immune responses [7,8]. Malignant tumours can modulate this balance by influencing macrophage subsets through a variety of mechanisms. Tumour cells can release chemokines like CSF-1 and IL-4, which induce macrophage transformation into M2-like polarised TAMs [9,10]. M2-like TAMs use their healing and growth inducing properties to promote tumour progression, angiogenesis and metastasis [11,12,13,14]. Simultaneously they suppress the initiation of an antitumoural immune response by the host [15,16,17]. With this in mind, it is not surprising that high numbers of TAMs are associated with a less favourable outcome in many forms of cancer, including breast cancer [18]. M1 macrophages on the other hand are considered to be a cytotoxic “anti-tumour” phenotype in the tumour microenvironment [19].

Our understanding of the association of TAMs and survival is currently limited and incomplete. A number of studies have demonstrated the unfavourable prognostic influence of M2-like TAMs [20,21], while more recent works have begun to emphasise the positive impact of M1-like TAMs on survival [22,23]. So far, there have been no reports on the combined influence of both macrophage phenotypes and long-term breast cancer progression.

To explore the effects of the two main macrophage phenotypes on breast cancer prognosis, we studied a carefully preselected and therefore homogenous cohort of patients whom participated in the accelerated partial breast irradiation (APBI) phase II trial at the University Hospital Erlangen [24]. M1 and M2 macrophage densities from pre-radiation therapy (RT) tumour resection samples were analysed and compared to disease-free survival (DFS) and in-breast recurrence-free survival (IBRFS). For the first time, we were able to create a prognostic model that combined cell densities of both macrophage phenotypes. This revealed new information on the use of TAMs as prognostic biomarkers for early breast cancer.

## 2. Results

**Figure 1:**
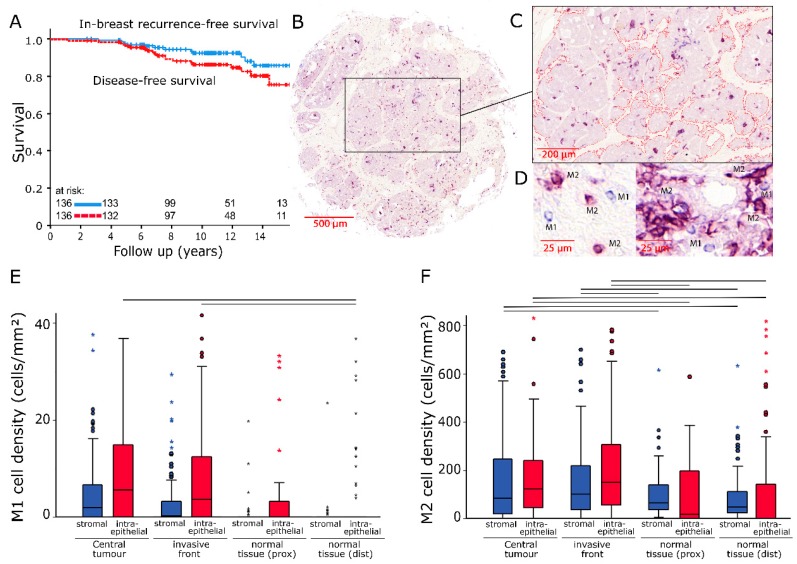
(**A**) In-breast recurrence-free survival and disease-free survival of the studied patient cohort analysed with the Kaplan–Meier method and log-rank test. (**B**) Scanned image of a representative breast cancer tissue microarray core (200× magnification). (**C**) Epithelial tumour compartment marked with an image analysis software. (**D**) Representative examples of M1 (IHC CD68+; blue) and M2 (IHC CD68+/CD163+; violet) tumour associated macrophages (TAMs). (**E**,**F**) Stromal and intraepithelial cell density distributions of M1 and M2 TAMs in samples from four different locations. The central line represents median values while the box is indicative of the interquartile range (IQR). Whiskers represent 1.5× IQR or minimum/maximum. Outliers are indicated by points (up to 3× IQR) or asterisks (>3 IQR). Black bars signify *p* < 0.05 in Student’s *t*-test.

In the early stage breast cancer cohort of 136 patients (Table 1) DFS was 85% while IBRFS was 92% after a 12 year follow up period (Figure 1A). Double immunostainings for CD68 and CD163 antigens were applied to pre-RT tissue samples from four locations: central tumour (CT), invasive front (IF), normal tissue taken from tumour proximity (prox) and normal tissue that was sampled distant from the tumour (dist). Numbers of infiltrating macrophages as well as the size of the intraepithelial and stromal compartments were quantified in each TMA core (Figure 1B). Subsequently in the stromal and intraepithelial compartments, cell densities for M1 and M2 polarised macrophages were calculated separately from four independent locations (Figure 1C). M1 macrophages were represented by cells that were exclusively positive for CD68. Cells that were positive for both CD68 and CD163 were considered to be M2 polarised (Figure 1D).

### 2.1. Cell Densities

Cell densities for M1 macrophages were relatively low, with the highest median values being 5.6 (standard deviation (SD): ±15 cells/mm^2^) and 3.7 cells/mm^2^ (SD: ±29.2 cells/mm^2^) in the intraepithelial compartment for CT and IF, respectively. Clear differences existed only between these two locations and normal tissue (dist) (*p* = 0.015 and *p* = 0.025) (Figure 1E). The CD163+ cell count in the tumour region revealed much higher cell densities in both the stromal (CT: Median/SD 83.6/±421.5 cells/mm^2^; IF: 100.4/±462.9 cells/mm^2^) and intraepithelial compartments (CT: Median/SD 121/±172.1 cells/mm^2^; IF: 148.6/±208.6 cells/mm^2^). In comparison, M2 macrophage densities in normal tissue were distinctly lower with stromal median values of 65.9 cells/mm^2^ (prox) and 45.6 cells/mm^2^ (dist) (both *p* < 0.05) (Figure 1F).

There was a moderate, positive monotonic correlation between M1 and M2 macrophage densities in normal tissue (dist: r = 0.478, *n* = 106, *p* < 0.001; prox: r = 0.409, *n* = 72, *p* < 0.001). In tumour samples from IF no strong correlations of this kind were found. Both macrophage phenotypes in CT were only weakly associated (r = 0.202, *n* = 119, *p* = 0.028) (Table 2).

### 2.2. Influence on Survival

**Figure 2:**
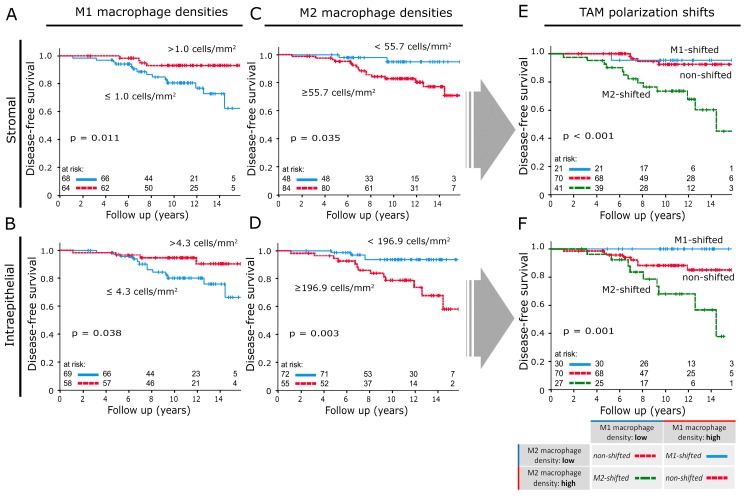
Disease-free survival analysed with the Kaplan–Meier method and log-rank test according to macrophage densities in invasive front samples: (**A**,**B**) M1 macrophage densities in the stromal and intraepithelial compartment. (**C**,**D**) M2 macrophage densities in the stromal and intraepithelial compartment. (**E**,**F**) Disease-free survival according to TAM polarisation shift classifications applied to the stromal and intraepithelial compartment of invasive front samples. The table located in the lower right section explains how results from (**A**–**D**) were combined to create the TAM shift classification.

High densities of M1 macrophages in IF (Figure 2A,B) and CT (Supplementary Figure S1) correlated with improved DFS and IBRFS (Supplementary Figure S2A,B) (*p* < 0.038). For M2 macrophages, the opposite was the case: high densities of CD163+ cells in the stromal and intraepithelial compartment of IF were associated with decreased DFS (Figure 2C,D) (*p* < 0.035) and IBRFS (Supplementary Figure S2A,B). M2 densities in both compartments of CT samples had similar associations with DFS (stromal: *p* = 0.02, intraepithelial: *p* = 0.122) (Supplementary Figure S1).

### 2.3. M-Shift Model

The individual M1 and M2 macrophage densities of each compartment and each tumour location were combined into three new subgroups. The subgroup allocations were based on whether samples belonged to the high or low cell density cohorts in the previously shown Kaplan–Meier plots (Figure 2A–D). Samples with low M2 and high M1 macrophage densities were classified as “M1-shifted”. “M2-shifted” was defined by high M2 and low M1 macrophage densities. If both phenotypes were classified as “high” or “low”, the sample was assigned to the “non-shifted” group.

### 2.4. Influence of M-Shifts on Survival

**Figure 3:**
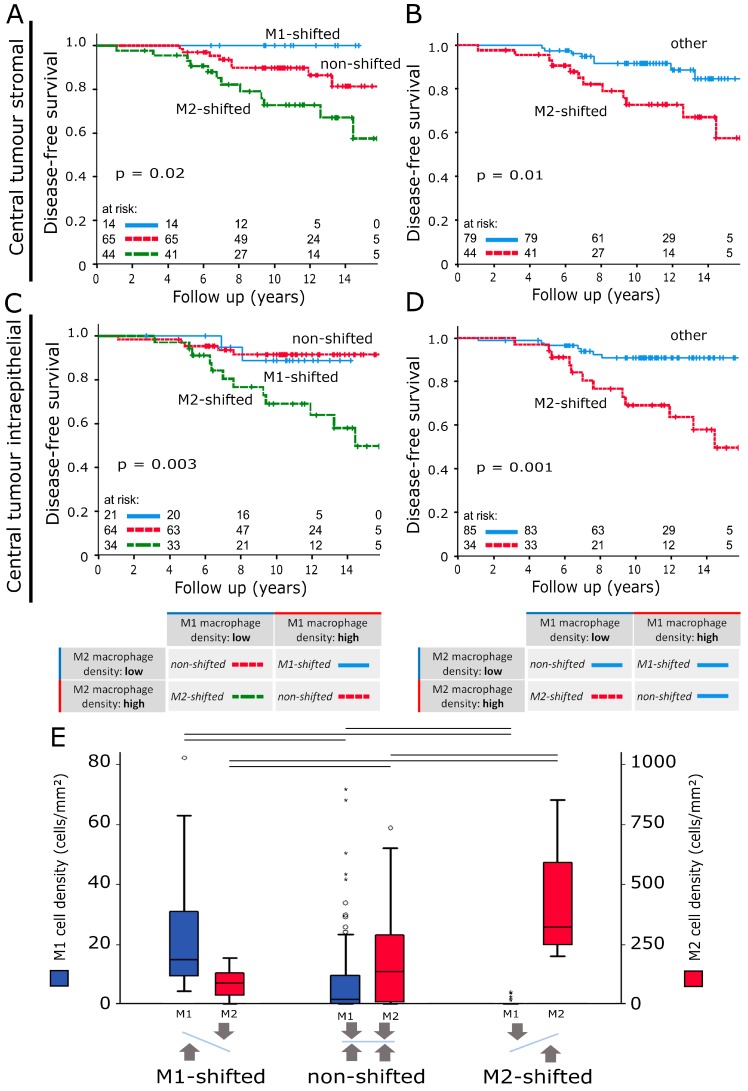
(**A**,**C**) Disease-free survival analysed with the Kaplan–Meier method and log-rank test according to TAM polarisation shift classifications based on stromal and intraepithelial macrophage densities in central tumour samples. (**B**,**D**) Versions of (**A**) and (**C**) that compare disease-free survival of the M2-shifted group to the remaining two groups. The tables below the survival plots give a detailed explanation of group compositions. (**E**) Intraepithelial macrophage density distributions in invasive front samples according to the TAM polarisation shift classification. The scale on the left indicates M1 macrophage densities (blue) while M2 cell densities can be found on the right (red). The central line represents median values while the box is indicative of the interquartile range (IQR). Whiskers represent 1.5× IQR or minimum/maximum. Outliers are indicated by points (up to 3× IQR) or asterisks (>3 IQR). Black bars signify *p* < 0.05 in Student’s *t*-test.

The stromal M-shift model at IF (Figure 2E) did not show a distinction between “M1-shifted” and “non-shifted” cases. Both groups exhibited a 12 yr DFS of over 90%. In comparison, the “M2-shifted” cohort showed an unfavourable prognosis with a dramatically reduced 12yr DFS of 68%. The M-shift model was a significantly better predictor of DFS than the respective macrophage phenotypes used as single markers (M1 and M2 showed a 12yr DFS of 77% and 80%, respectively). The intraepithelial model resulted in three survival groups (Figure 2F). The “M1-shifted” group had a 12 yr DFS of 100%, while the “M2-shifted” cohort had a 12 yr DFS of only 68%. “Non-shifted” cases settled in between those two groups and had a 12 yr DFS of 85%. The M-shift model was again better than macrophage counts alone at predicting recurrence or metastasis (M1: 12 yr DFS 80%; M2: 12 yr DFS: 74%). Samples from CT had comparable prognostic power (Figure 3A,C) but overall macrophage shifts at IF correlated more strongly with DFS.

To make this combined model more comparable to other parameters, it was simplified into two prognostic groups. “M2-shifted” remained while “M1-shifted” and “non-shifted” were merged (Figure 3B,D; Supplementary Figure S3A,B). Table 3 summarises group sizes, 12 yr DFS and IBRFS and *p*-values. A model that was based on M1/M2 cell density ratios was also explored, but not included as it was less accurate at predicting DFS. Comparing the IF intraepithelial M-shift model subgroups, statistically significant differences in M1 and M2 macrophage cell densities were observed (*p* < 0.05) (Figure 3E). M2 densities were at the lowest in “M1-shifted” and this value rose from “non-shifted” to “M2-shifted”. For M1 macrophages the opposite was the case, with “M1-shifted” registering the highest cell densities. Stromal cell distributions did not differ (Supplementary Figure S3C,D). Multivariate analyses revealed the stromal (*p* = 0.01) and intraepithelial TAM shift classification at IF (*p* = 0.039) to be independent risk factors for DFS (Table 4).

**Figure 4:**
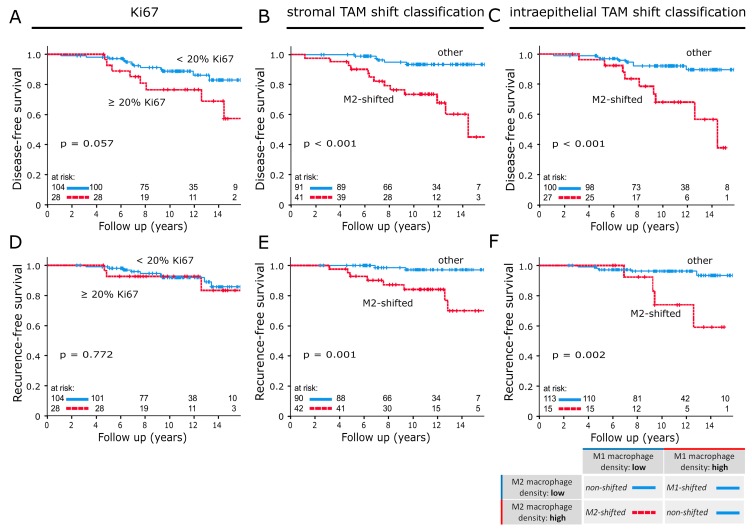
Disease-free survival analysed with the Kaplan–Meier method and log-rank test according to (**A**) Ki67 and (**B**,**C**) the TAM shift classification applied to invasive front samples. In-breast recurrence-free survival analysed with the Kaplan–Meier method and log-rank test according to (**D**) Ki67 and (**E**,**F**) the TAM shift classification applied to invasive front samples. In (**B**,**C**,**E**,**F**), M2-shifted was compared to the remaining two groups of the TAM shift classification. The table below the survival plots gives a detailed explanation of group compositions.

The proliferation index Ki67 was the strongest conventional prognostic parameter in this study population. In a direct comparison, the M-shift model was superior at predicting DFS and IBRFS. (Figure 4A–F). No statistically significant correlations between macrophage shift classifications and clinicopathological characteristics were discovered (Table 5).

## 3. Discussion

Previous studies have shown a direct association between macrophage infiltration in breast cancer and increased mortality [20,22,23,25,26]. Whereas these studies looked at patients with various stages of breast cancer, our objective was to explore the value of macrophage densities as an independent prognostic biomarker in early stage hormone receptor-positive (HR+) breast cancer. We analysed tissue from 136 patients who participated in a prospective APBI phase II trial at the Universitätsklinikum Erlangen [24,27]. This trial had strict inclusion criteria and only recruited patients with early stage HR+ breast cancer, thus resulted in a very uniform set of clinicopathological characteristics. Thereby, providing us with the opportunity to study the influence of macrophage densities on survival with minimal confounding factors.

Samples from these patients’ tumours were double stained for CD68 and CD163. CD68 is regarded as a pan-macrophage marker which is expressed by both M1 and M2 macrophages [28]. CD163 is a scavenger receptor, predominantly found in M2 or M2-like macrophages [29]. Immunostaining of CD68 highlighted all macrophages. These macrophages were simultaneously subjected to CD163 immunostaining. Hence, macrophages that were CD68 positive and CD163 negative were considered M1 polarised and ones that were positive for both markers represented M2 and M2-like polarised TAMs. This is an important distinction from previous studies that only marked for CD68 or applied CD68 and CD163 immunostainings separately.

Scans of stained samples were analysed with the help of an image analysis software. We assessed the size of the stromal and intraepithelial compartments as well as the number of CD68+ and CD163+ cells. As expected, we found higher numbers of macrophages in tumour vicinity than in normal breast tissue. This can be seen as evidence of local inflammation at the tumour site. Intratumorally, we found that high numbers of M2 macrophages in the stromal and intraepithelial compartment had a negative association with IBRFS and DFS. In contrast, high cell densities of M1 macrophages were associated with improved IBRFS and DFS. These results are consistent with the outcome of multiple studies. In ovarian and non-small cell lung cancer cytotoxic M1 macrophages are associated with a favourable prognosis [30,31]. In pancreatic and gastric cancer, immunosuppressant M2 polarised macrophages promote tumour growth and decrease survival [32,33]. Specifically for breast cancer, Honkanen et al., and Jeong et al. have recently published similar findings in study populations with more advanced disease stages [22,23].

Throughout this study, it has come to light that it is insufficient to view macrophage phenotypes as isolated factors influencing survival in breast cancer, as it is well established that they represent opposite ends of a transformative spectrum of the same cell type [34]. In healthy tissue, a delicate balance is upheld between the pro-inflammatory functions of M1 macrophages and the repair and maintenance functions of M2 macrophages [8]. Malignant tumours break this balance by recruiting macrophages through chemokines like M-CSF and IL-4, leading to a transformation into M2-like polarised TAMs [9,10]. Results of this study support this theory with distinct correlations between the two macrophage phenotype densities found in normal breast tissue. Concurrently these correlations were mostly absent in tumour samples, showing that the physiological balance is altered in neoplastic tissue.

Based on this hypothesis, we created three tumour classifying subgroups including the combined information on M1 and M2 TAM densities. Instead of using total numbers, the focus of this classification was on the polarisation status of the TAM population as a whole. Changes in the ratio of TAM phenotypes were described as shifts. A shift is defined by high densities of one TAM type with simultaneous low densities of its counterpart. A fitting analogy for this process would be a scale losing its balance and tipping over to one side.

Hypothetically, “M1-shifted” represents a cytotoxic immune response. The tumour is not able to recruit a sufficient number of M2-like TAMs and is instead recognized and attacked by high numbers of M1 macrophages. This disruption of balance into the direction of cytotoxicity is the best-case scenario for patient survival and entails a very good prognosis. “Non-shifted” describes tumours which do not change the normal ratio of macrophage phenotypes. They are characterized by high or low densities of both macrophage activation states. Our results showed that these two constellations have an almost identically favourable prognosis and can therefore be classified as one prognostic group (Supplementary Figure S3E,F). One might presume that “non-shifted” tumours can neither recruit enough macrophages for an M2 shift nor do they get targeted by an M1 response. It is unclear why TAM populations in these cases do not perform a shift. A possible explanation could be a lack of chemokine release for TAM recruitment. Our most significant finding concerning DFS was in the “M2-shifted” group. These tumours have taken full control of the TAM population and educated them towards M2 polarisation. Almost no cytotoxic M1 macrophages remain and high numbers of M2-like TAMs suppress the immune system, promote tumour growth, angiogenesis and metastasis [12,13,14,15]. The macrophage polarisation shift classification could identify high-risk patients more accurately than any conventional biomarker or prognostic parameter available to us in this study (Figure 4).

Potential implications of these findings for macrophages as a predictive marker are immense. The sole use of one macrophage phenotype should be avoided as results will be weakened by the “non-shifted” group. TAM densities of one type only show their full potential as predictors of significantly reduced DFS if they occur in the right combination with their counterparts. If such a combined classification results in an “M2-shifted” TAM population, the tumour belongs to a high-risk group and might be a candidate to receive extended therapy. Additional non-radiation based treatment might be appropriate, as there is evidence of macrophage induced radio-resistance after TAM cancer cell fusion [35,36]. Waks et al. also reported a possible role for M2-like macrophages in chemo-resistance in HR+ breast cancer [37]. These results demonstrate that macrophage polarisation targeting therapies like CSF inhibitors might become valuable therapeutic options for breast cancer treatment [38,39].

Another finding of this study was that data from IF samples generally had the highest prognostic value. Nonetheless, the M-shift model consistently predicted DFS and IBRFS in both tumour locations and compartments. This indicates that the M-shift model is reliable and reproducible.

In the face of these results, it needs to be emphasized that this study was performed on a very uniform low-risk breast cancer cohort that was characterized by HR positivity, small tumour size and no metastasis. Until studies with a comparable setup and method are performed on a larger number and wider range of breast cancer types, we cannot conclude whether the observed very strong association between macrophage shifts and DFS is restricted to early breast cancer.

## 4. Materials and Methods

### 4.1. Breast Cancer Patients and Clinical Data

A total of 136 patients were treated for early stage breast cancer at the Universitätsklinikum Erlangen between 2000 and 2005 as part of a prospective APBI phase II trial. APBI was performed with interstitial multicatheter brachytherapy. Inclusion criteria of this study were histopathologically assured invasive breast carcinoma of any histology of ≤3 cm in diameter, unifocal and unicentric breast cancer, clear resection margins of at least 2 mm in any direction, hormone sensitivity (ER+/PR+, ER+/PR−, ER−/PR+), histologic grading of 1 or 2, no lymph vessel and no blood vessel invasion, pN0/pNmi, no distant metastases and age ≥35 years [24,27]. All patients received breast conserving surgery and brachytherapy. Additionally, 115 patients (84.6%) were treated with hormonal therapy, two (1.5%) with chemotherapy and seven (5.1%) received both treatments. Clinical data and pathological characteristics that were prospectively collected during the APBI study can be found in Table 1. Tissue samples were obtained from tumour resections prior to RT. After a 12 year follow up period, breast cancer specific survival was 99%. Disease-free survival (DFS), which was defined as survival without local recurrence or distant metastasis, was 85% while the in-breast recurrence-free survival rate was 92% (Figure 1A). Two out of a total of 12 in-breast recurrences were exclusively classified as in-field recurrences. The remaining 10 recurrences were characterized by diffuse infiltration of the whole breast or in-field/out-of-field classification was not possible.

Written informed consent was obtained “front door” from all patients for collection of their tissue and clinical data. The use of formalin fixed paraffin-embedded material from the Archive of the Institute of Pathology was approved by the Ethics Committee of the Friedrich-Alexander-University of Erlangen-Nuremberg on 24 January 2005 (21_ 19 B), waiving the need for consent for using the existing archived material.

### 4.2. TMA Construction and Immunohistochemistry

Four tissue microarrays (TMAs) each with a diameter of 2mm per core were processed from 136 paraffin-embedded tumour resections (Figure 1B). Samples were taken from the central tumour (CT), the invasive front (IF), normal tissue in tumour proximity (prox) and normal tissue distant from the tumour (dist). Then, 2 µm tissue sections were de-paraffinised in xylene and rehydrated with graded ethanol. Double staining for CD68 and CD163 was performed using the Alkaline phosphatase detection kit (POLAP-100, Zytomed Systems GmbH, Berlin, Germany) with Fast red (Sigma-Aldrich, Deisenhofen, Germany) as chromogen for CD163 and Fast blue (Sigma-Aldrich) as chromogen for CD68 detection [40].

Stained TMAs were scanned with a high throughput scanner (Mirax Scan, Zeiss, Göttingen, Germany). The software Pannoramic Viewer (3D Histech, Budapest, Hungary) was used for further processing of the scans. In 132 out of 136 IF samples, stromal tissue could be identified (97%) while 127 contained epithelium (93%). For biopsies from CT, these numbers were 123 (90%) and 119 (88%), respectively. The majority of these discrepancies can be explained by samples which only consisted of stromal or epithelial tissue. Another factor to account for this discrepancy was loss of material during the staining process.

Biopsies of normal breast tissue often contained very little tissue or mostly fat. In normal tissue (prox) samples, cells could be counted in the stromal compartment in 93 (68%) cases while 72 (53%) contained epithelium. For normal tissue (dist) these numbers were 116 (85%) and 96 (71%), respectively. Intrinsic breast cancer subtypes were classified according to the definitions released by the St Gallen International Expert Consensus on the Primary Therapy of Early Breast Cancer [41]. The cut-off point for Ki67 was based on results by Fasching et al., which described Ki67 expression levels ≥20% as an unfavourable prognostic factor in early breast cancer [42].

### 4.3. Quantification of Macrophages

Image processing software (Biomas, Erlangen, Germany) was used to quantify cell densities. Stromal and intraepithelial compartments were marked (Figure 1C) and their respective areas registered. Macrophage numbers were counted semi-automatically. Inclusion criteria were size, morphology and colour (Figure 1D). Cell densities were calculated for both compartments separately.

### 4.4. Statistical Analyses

SPSS version 26 (IBM Inc., Chicago, IL, USA) was used for all statistical analyses. Spearman’s Rho and Fisher’s exact test were used to find correlations. Mean values of cell densities in different tumour locations and subgroups were compared using Student’s *t*-test for independent samples and Welch’s test. Optimal cut-off points for macrophage density groups (low vs high) were determined for disease-free survival (DFS) and in-breast recurrence-free survival (IBRFS) through receiver operating characteristic (ROC) curve analysis. Survival curves for DFS and IBRFS were plotted using the Kaplan–Meier method and compared with the log-rank test. Cox proportional hazards model was used to calculate hazard ratios of macrophage cell densities and clinicopathological characteristics. Covariates with *p* < 0.25 in univariate analysis were included in multivariate Cox regression. The proportional hazards assumption was tested by visual inspection of log minus log curves and was found to be satisfactory for all multivariate covariates. *p*-values < 0.05 were considered to be statistically significant.

## 5. Conclusions

In early breast cancers, high densities of M2 macrophages and low densities of M1 macrophages in the tumour epithelium and stroma are strongly associated with reduced DFS. Our findings revealed the importance of examining both macrophage phenotypes together and in context of their relative frequencies. A proposed classification based on TAM polarisation shifts identified a high-risk group of “M2-shifted” tumours that are unlikely to fully respond to accelerated partial breast irradiation. The M-shift model might aid clinicians in selecting breast cancer patients in need of extended therapy.

## Figures and Tables

**Table 1:** Clinical characteristics.

Variables	Groups					
Age (years)	Mean: 59.1;	<50: 27 (19.9%);				≥50: 109 (80.1%)
T category	pT1mic: 6 (4.4%)	pT1a: 8 (5.9%)	pT1b: 33 (24.3%)		pT1c: 79 (58.1%)	pT2: 10 (7.4%)
N category	N0: 133 (97.8%)	N1: 3 (2.2%)				
Stage	UICC I: 124 (91.2%)	UICC II: 12 (8.8%)				
Tumour size (mm)	<10: 34 (25%);	10–20: 92 (67.6%);				>20: 10 (7.4%)
Histological grading	G1: 36 (26.5%)	G2: 93 (68.4%)		G3: 4 (2.9%)		n.a. 3 (2.2%)
Histological typing	Lobular: 20 (14.7%)	No special type: 95 (69.9%)				other: 21 (15.4%)
Ki67	<20: 104 (76.5%)	≥20: 28 (20.6%)				n.a. 4 (2.9%)
Oestrogen receptor status	Positive: 131 (96.3%)	Negative: 1 (0.7%)				n.a. 4 (2.9%)
Progesterone receptor status	Positive: 122 (89.7%)	Negative: 11 (8.1%)				n.a. 3 (2.2%)
Her2 status	Positive: 8 (5.9%)	Negative: 124 (91.2%)				n.a. 4 (2.9%)
Subtype	Luminal A: 89 (65.4%)	Luminal B: 41 (30.1%)				n.a. 6 (4.4%)
Hormone therapy	Yes: 122 (89.7%)	No: 14 (10.3%)				
Chemotherapy	Yes: 9 (6.6%)	No: 127 (93.4%)				

n.a. = not available.

**Table 2:** Correlations between macrophage phenotypes in different tumour compartments.

M1 Groups	Indicators	M2 Cell Densities Central Tumour	M2 Cell Densities Invasive Front	M2 Cell Densities Normal Tissue (Prox)	M2 Cell Densities Normal Tissue (Dist)
M1 cell densities central tumour	Correlation coefficient	0.202 *	0.095	−0.020	0.090
	*p*	**0.028**	0.322	0.872	0.412
	*n*	**119**	111	68	86
M1 cell densities invasive front	Correlation coefficient	0.121	0.100	0.133	0.110
	*p*	0.205	0.262	0.273	0.296
	*n*	111	127	70	92
M1 cell densities normal tissue (prox)	Correlation coefficient	−0.097	0.100	0.409 **	0.399 **
	*p*	0.429	0.409	**<0.001**	**0.001**
	*n*	68	70	**72**	**65**
M1 cell densities normal tissue (dist)	Correlation coefficient	−0.031	0.137	0.263 *	0.478 **
	*p*	0.778	0.193	**0.034**	**<0.001**
	*n*	86	92	**65**	**106**

* *p* ≤ 0.05, ** *p* ≤ 0.001.

**Table 3:** Summary of 12-year survival according to Ki67, macrophage densities and macrophage shift classifications. Cut-off points for low vs. high macrophage densities for disease-free survival and in-breast recurrence-free survival can be found in the respective Kaplan–Meier plots.

Breast Cancer	Disease-Free Survival			In-Breast Recurrence-Free Survival		
Variables	*N*	12 yr DFS (%)	*p* (Log Rank)	*N*	12 yr IBRFS (%)	*p* (Log Rank)
Ki67						
<20%	104	86	0.057	104	92	0.772
≥20%	28	77		28	93	
Central tumour stromal						
M1 low	53	78	0.095	51	88	0.241
vs. M1 high	70	87		72	94	
M2 low	23	100	**0.02**	31	100	**0.033**
vs. M2 high	100	79		92	89	
M1-shifted + non-shifted	79	89	**0.01**	85	95	**0.033**
vs. M2-shifted	44	73		38	84	
Central tumour intraepithelial						
M1 low	63	75	**0.035**	61	86	0.094
vs. M1 high	56	90		58	96	
M2 low	50	90	0.122	54	96	0.132
vs. M2 high	69	78		65	88	
M1-shifted + non-shifted	85	91	**0.001**	87	96	**0.004**
vs. M2-shifted	34	64		32	77	
Invasive front stromal						
M1 low	68	77	**0.011**	70	88	**0.011**
vs. M1 high	64	93		62	98	
M2 low	48	95	**0.035**	48	97	0.101
vs. M2 high	84	80		84	91	
M1-shifted + non-shifted	91	93	**<0.001**	90	97	**0.001**
vs. M2-shifted	41	68		42	84	
Invasive front intraepithelial						
M1 low	69	80	**0.038**	57	86	**0.025**
vs. M1 high	58	90		70	99	
M2 low	72	94	**0.003**	95	96	**0.004**
vs. M2 high	55	74		32	85	
M1-shifted + non-shifted	100	90	**<0.001**	113	96	**0.002**
Vs. M2-shifted	27	68		15	74	

**Table 4:** Univariate and multivariate analysis of disease-free survival according to Cox’s proportional hazards model. Parameters marked with an asterisk were not included in calculations because group sizes were not comparable.

Breast Cancer	Univariate Analysis			Multivariate Analysis		
Variable	Hazard Ratio	95% C.I.	*p*	Hazard Ratio	95% C.I.	*p*
Age (yr) (<50 (*n* = 26) vs. ≥50 (*n* = 91))	1.757	0.352–8.765	0.492	---	---	---
pT category (pT1 (*n* = 108) vs. pT2 (*n* = 9))	1.346	0.312–5.809	0.691	---	---	---
Stage (UICC I (*n* = 107) vs. UICC II (*n* = 10))	1.892	0.553–6.47	0.309	---	---	---
Tumour size (mm) (<20 (*n* = 102) vs. ≥20 (*n* = 15))	1.109	0.044–27.889	0.950	---	---	---
Histological grading (G1 (*n* = 33) vs. G2-3 (*n* = 84))	1.492	0.304–7.318	0.622	---	---	---
Histological typing (non-lobular (*n* = 101) vs. lobular (*n* = 16))	0.536	0.089–3.22	0.495	---	---	---
ER status (negative (*n* = 1) vs. positive (*n* = 116))	*	*	*	---	---	---
PR status (negative (*n* = 11) vs. positive (*n* = 106))	1.152	0.065–20.312	0.923	---	---	---
Her2 status (negative (*n* = 110) vs. positive (*n* = 7))	1.357	0.137–13.424	0.794	---	---	---
Ki67 (<20 (*n* = 104) vs. ≥20 (*n* = 28))	2.332	0.95–5.725	0.065	1.963	0.712–5.41	0.193
Hormone therapy (No (*n* = 12) vs. Yes (*n* = 105))	2.042	0.166–25.048	0.577	---	---	---
Chemotherapy (No (*n* = 109) vs. Yes (*n* = 8))	3.831	0.374–39.207	0.258	---	---	---
TAM shift classification intraepithelial (other (*n* = 93) vs. M2-shifted (*n* = 24))	4.796	1.846–12.461	0.001	2.890	1.058–7.896	0.039
TAM shift classification stromal (other (*n* = 81) vs. M2-shifted (*n* = 36))	9.359	2.399–36.509	0.001	4.223	1.408–12.667	0.010

ER = oestrogen receptor; PR = progesterone receptor.

**Table 5:** Correlations between clinicopathological characteristics and macrophage shifts in both tumour compartments of invasive front samples.

Breast Cancer			Stromal TAM Shift Classification (*n* = 132)			Intraepithelial TAM Shift Classification (*n* = 127)		
Characteristics	Group	*n* (total)	M1-Shifted + Non-Shifted	M2-Shifted	*p*	M1-Shifted + Non-Shifted	M2-Shifted	*p*
Age (yr)	<50	27	21 (23%)	6 (15%)	0.353	21 (21%)	6 (22%)	1.00
	≥50	109	70 (77%)	35 (85%)		79 (79%)	21 (78%)	
T Category	pT1	126	86 (95%)	36 (88%)	0.284	94 (94%)	24 (89%)	0.399
	pT2	10	5 (5%)	5 (12%)		6 (6%)	3 (11%)	
Stage	UICC I	124	86 (95%)	35 (85%)	0.095	93 (93%)	24 (89%)	0.442
	UICC II	12	5 (5%)	6 (15%)		7 (7%)	3 (3%)	
Tumour size (mm)	<20	120	82 (90%)	34 (83%)	0.259	90 (90%)	22 (82%)	0.31
	≥20	6	9 (10%)	7 (17%)		10 (10%)	5 (18%)	
Histological grading	G1	36	27 (31%)	9 (24%)	0.52	30 (31%)	4 (17%)	0.208
	G2+G3	93	60 (69%)	29 (76%)		66 (69%)	20 (83%)	
	n.a.	7						
Histological typing	Non lobular	116	78 (86%)	35 (85%)	1.00	87 (87%)	23 (85%)	0.758
	Lobular	20	13 (14%)	6 (15%)		13 (13%)	4 (15%)	
DCIS	No	68	44 (54%)	21 (55%)	1.00	47 (52%)	16 (67%)	0.25
	Yes	56	38 (46%)	17 (45%)		44 (48%)	8 (33%)	
	n.a.	12						
Ki67	<20	100	71 (82%)	29 (71%)	0.176	78 (81%)	19 (70%)	0.285
	≥20	28	16 (18%)	12 (29%)		18 (19%)	8 (30%)	
	n.a.	4						
ER status	Neg	1	1 (1%)	0	1.00	1 (1%)	0	1.00
	Pos	131	86 (99%)	41 (100%)		95 (99%)	27 (100%)	
	n.a.	4						
PR status	Neg	11	9 (10%)	2 (5%)	0.501	7 (7%)	4 (15%)	0.253
	Pos	122	79 (90%)	39 (95%)		90 (93%)	23 (85%)	
	n.a.	3						
Her2 status	Neg	124	82 (94%)	38 (93%)	0.71	89 (92%)	26 (100%)	0.201
	Pos	8	5 (6%)	3 (7%)		8 (8%)	0	
	n.a.	4						
Subtype	Luminal A	89	59 (68%)	26 (65%)	0.688	65 (69%)	17 (63%)	0.641
	Luminal B	41	27 (31%)	14 (35%)		29 (31%)	10 (37%)	
	n.a.	6						

n.a. = not available; ER = oestrogen receptor; PR = progesterone receptor.

## Supplementary Materials

The following are available online at https://www.mdpi.com/2072-6694/12/2/446/s1, **Figure S1:** Disease-free survival analysed with the Kaplan–Meier method and log-rank test according to macrophage densities in central tumour samples. Macrophage phenotype and compartment are indicated by descriptions next to the black bars. **Figure S2:** (A) In-breast recurrence-free survival analysed with the Kaplan–Meier method and log-rank test according to macrophage densities in invasive front samples. (B) In-breast recurrence-free survival according to macrophage densities in central tumour samples. Macrophage phenotype and compartment are indicated by descriptions next to the black bars. **Figure S3:** In-breast recurrence-free survival analysed with the Kaplan–Meier method and log-rank test according to (A) stromal and (B) intraepithelial TAM shift classifications in central tumour samples. (C,D) Stromal and intraepithelial macrophage density distributions in invasive front samples according to the intraepithelial TAM polarisation shift classification. The central line represents median values while the box is indicative of the interquartile range (IQR). Whiskers represent 1.5× IQR or minimum/maximum. Outliers are indicated by points (up to 3× IQR) or asterisks (>3 IQR). Black bars signify *p* < 0.05 in Student’s *t*-test. (E,F) Disease-free survival according to non-shifted groups of the TAM shift classification applied to invasive front samples. The tables below the survival plots give a detailed explanation of group compositions.
