# Supplementary figures and images for: Accelerated Partial Breast Irradiation: Macrophage Polarisation Shift Classification Identifies High-Risk Tumours in Early Hormone Receptor-Positive Breast Cancer
Source: Cancers (Basel). 2020 Feb 14;12(2):446. doi: 10.3390/cancers12020446 (PMC7072550; doi:10.3390/cancers12020446)

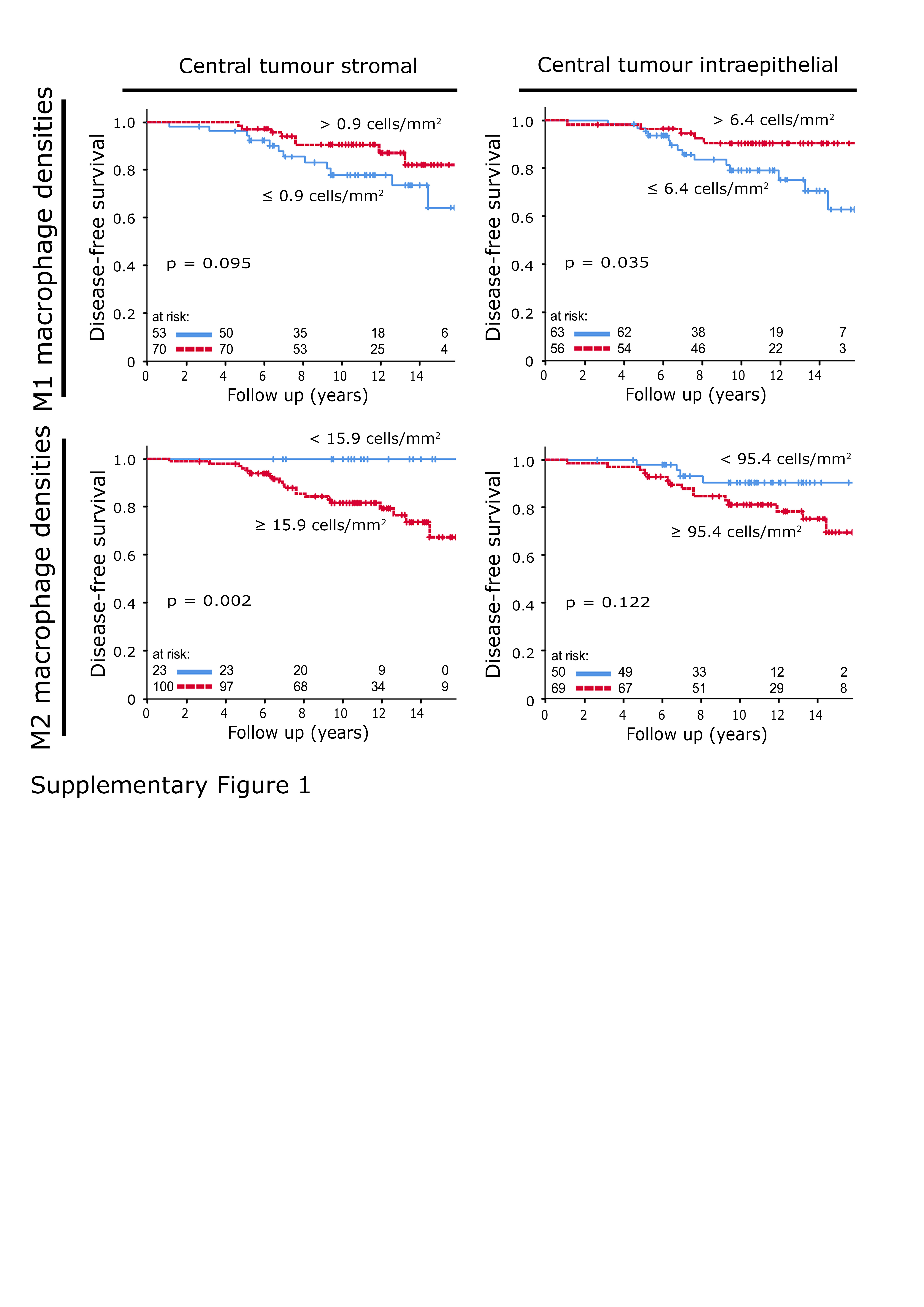

Supplement: Supplementary file 1 [file cancers-12-00446-s001.zip › cancers-707456-supplemetary/Supplement Figure S1.jpg]

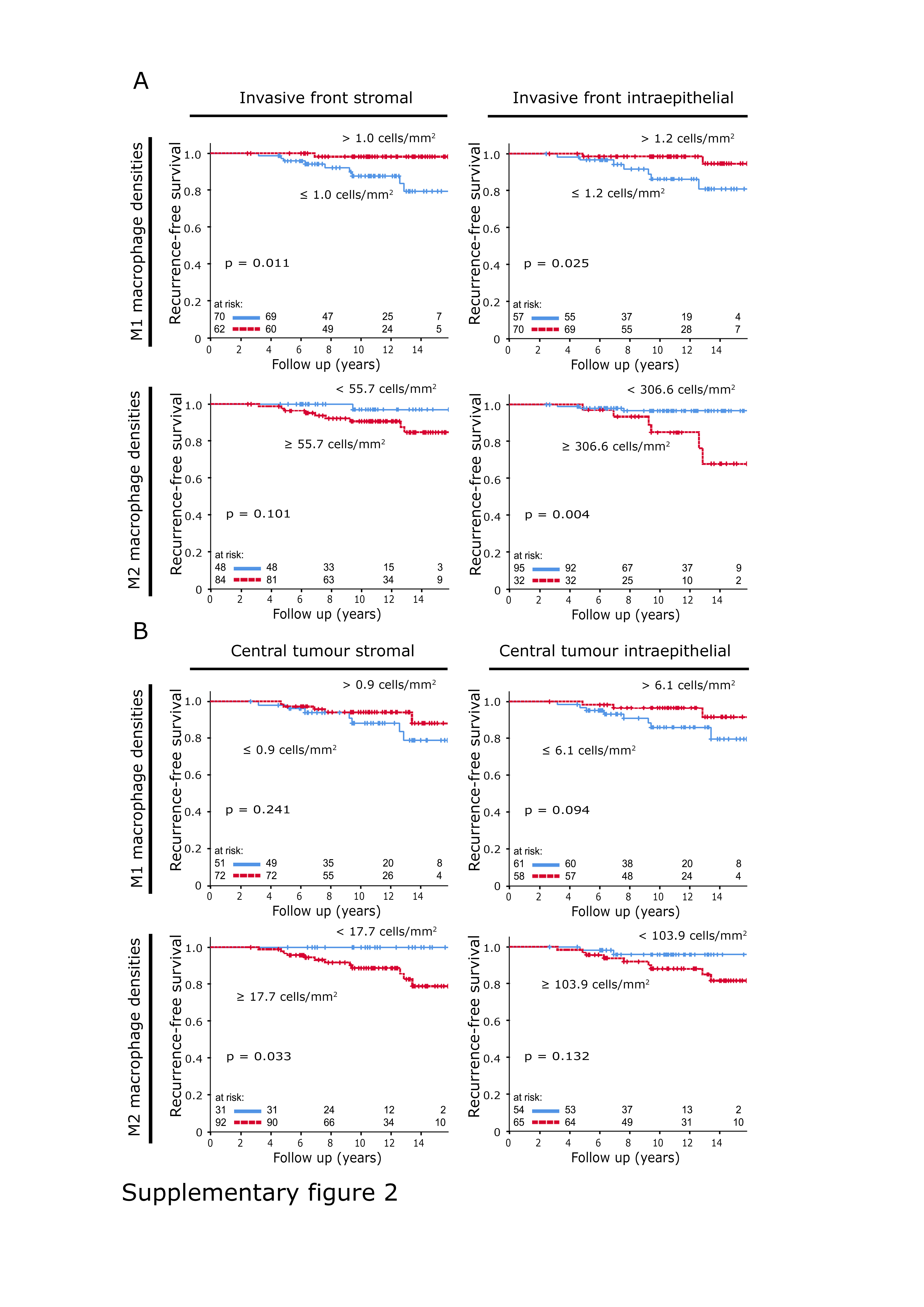

Supplement: Supplementary file 1 [file cancers-12-00446-s001.zip › cancers-707456-supplemetary/Supplement Figure S2.jpg]

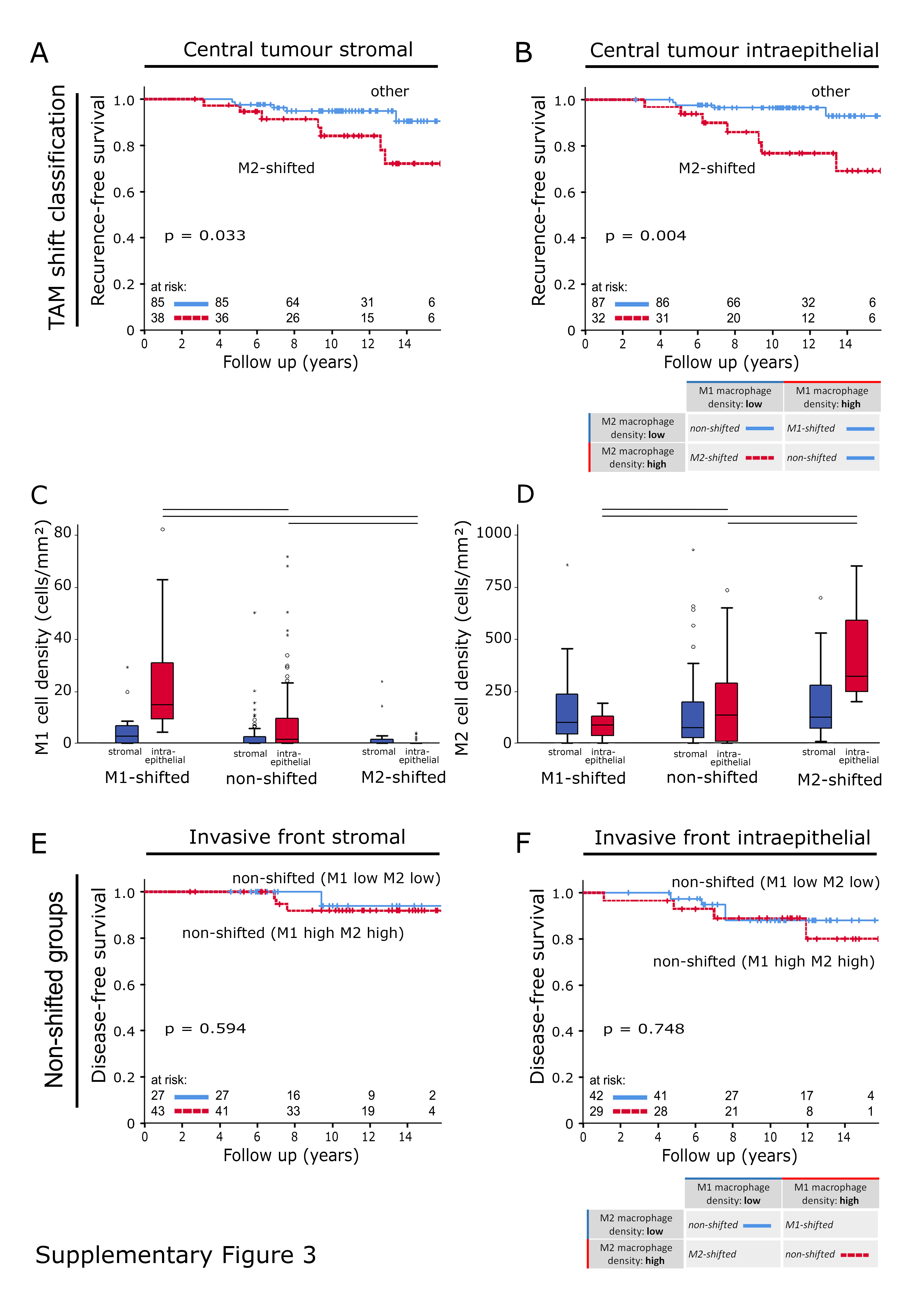

Supplement: Supplementary file 1 [file cancers-12-00446-s001.zip › cancers-707456-supplemetary/Supplement Figure S3.jpg]
